# Supplementary material for: Characterizing SARS-CoV-2 mutations in the United States
Source: Res Sq. 2020 Aug 11:rs.3.rs-49671. Preprint. [Version 1] doi: 10.21203/rs.3.rs-49671/v1 (PMC7430589; doi:10.21203/rs.3.rs-49671/v1)
Supplement: Supplement [file 2019nCoVEvolutionSupportingInformation.pdf]

Supporting Information
Characteristics of SARS-CoV-2 mutations in the United States

Rui Wang[1], Jiahui Chen[1], Kaifu Gao[1], Yuta Hozumi [1], Changchuan Yin[2], and Guo-Wei Wei[1,3,4*]
[1] Department of Mathematics,
Michigan State University, MI 48824, USA.
[2] Department of Mathematics, Statistics, and Computer Science,
University of Illinois at Chicago, Chicago, IL 60607, USA
[3] Department of Electrical and Computer Engineering,
Michigan State University, MI 48824, USA.
[4] Department of Biochemistry and Molecular Biology,
Michigan State University, MI 48824, USA.

July 22, 2020

Contents

S1 Supplementary Figures **1**
S1.1 K-Means clustering 1
S1.2 Proteoforms 1

S2 Supplementary Tables **2**

*Corresponding author. E-mail: weig@msu.edu

S1 Supplementary Figures

S1.1 K-Means clustering

The k-means clustering is used to classify the SARS-CoV-2 the SNP variants. The Elbow method is used to determine the optimal number of clusters. Our results demonstrate four main clusters in the United States (US) as shown in Figure S1, which plots the within-cluster sum of squares according to the number of clusters k for the SNP variants in the United States based on Jaccard distance metric. The optimal values of k-mean clusters is shown as the turning point in the in the elbow plots.

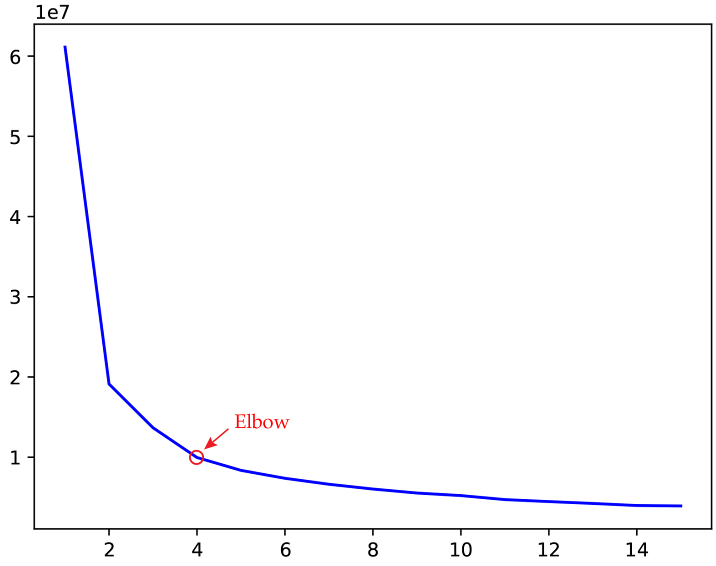

Figure S1: The plot of WCSS according to the number of clusters based on Jaccard distance metric. Here, Jaccard distance-based representation is taken as the input feature. The arrows point out the optimal number of clusters. The within-cluster sum of squares against the number of clusters for the SNP variants in the United States. The optimal number of clusters in the United States is four.

S1.2 Proteoforms

Figure S2 shows the visualization of the proteoforms of SARS-CoV-2 NSP2, NSP13, NSP12, spike protein, ORF8, and ORF3a. The top 8 mutations are marked in coor. The red color represents the wild type residue and the yellow color represents the mutant type.

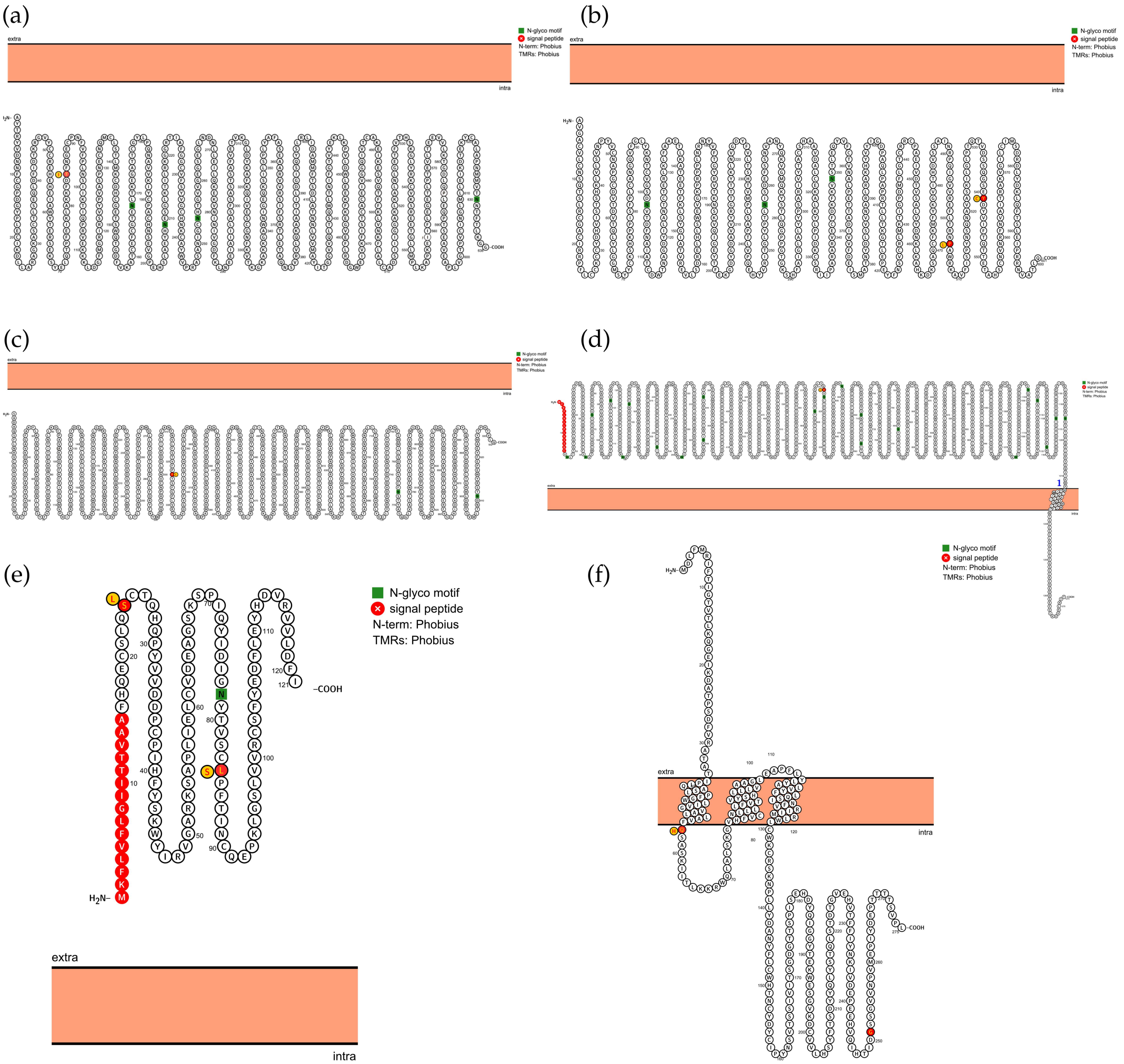

Figure S2: The visualization of six SARS-CoV-2 proteoforms. (a) Proteoform of NSP2. (b) Proteoform of NSP13. (c) Proteoform of NSP12. (d) Proteoform of spike protein. (e) Proteoform of ORF8. (f) Proteoform of ORF3a. The red color represents the wild type and the yellow represents the wild type.

S2 Supplementary Tables

Total 8 spreadsheets are merged in the Supporting_Tables.xlsx.

Table S1: S1_snpRecords_07142020_US: The SNP profiles in the United States. (Up to July 14, 2020).

Table S2: Acknowledgment table provided by GISAID in Jan 2020.

Table S3: Acknowledgment table provided by GISAID in Feb 2020.

Table S4: Acknowledgment table provided by GISAID in March 2020.

Table S5: Acknowledgment table provided by GISAID in April 2020.

Table S6: Acknowledgment table provided by GISAID in May 2020.

Table S7: Acknowledgment table provided by GISAID in June 2020.

Table S8: Acknowledgment table provided by GISAID in July 2020.
