# Supplementary figures and images for: Characterizing SARS-CoV-2 mutations in the United States
Source: Res Sq. 2020 Aug 11:rs.3.rs-49671. Preprint. [Version 1] doi: 10.21203/rs.3.rs-49671/v1 (PMC7430589; doi:10.21203/rs.3.rs-49671/v1)

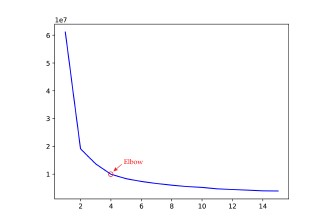

Supplement: Supplement [file FigureS1.jpg]

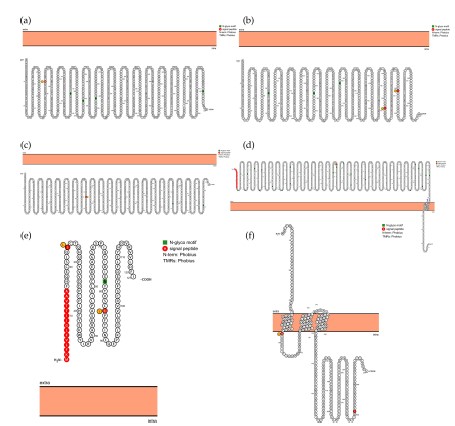

Supplement: Supplement [file FigureS2.jpg]
